# Aspartate aminotransferase-platelet ratio index, adiponectin and body mass index in children with fatty liver

**DOI:** 10.1186/1687-9856-2013-S1-P107

**Published:** 2013-10-03

**Authors:** Ninung RD Kusumawati, Maria Mexitalia, Suci Romadhona, Agustini Utari

**Affiliations:** 1 Department of Pediatric, Faculty of Medicine, Diponegoro University / Dr. Kariadi Hospital, Semarang, Indonesia

## 

Obesity in children is becoming a global epidemic. Non-alcoholic fatty liver disease (NAFLD) is a highly prevalent and potentially serious complication of childhood obesity. Adiponectin level was decreased on obese children. Adiponectin is a protective factor against non-alcoholic fatty liver disease on obesity. The early identification of fibrosis is important in children with NAFLD in order to prevent the development of liver disease in adulthood. One of non-invasive procedure to predict liver fibrosis is the aspartate aminotransferase (AST)-platelet ratio index (APRI). The purpose of our study was to assess a correlation between APRI, adiponectin and body mass index (BMI) in obese children with fatty liver.

A cross-sectional study was conducted from August to September 2007. Subjects were obese children from one junior high school in Semarang, Indonesia. Complete blood count, transaminase enzyme measurement, adiponectin level and abdominal ultrasound (USG) were performed on each subject. Only subjects with bright liver on USG underwent APRI analysis. Spearman’s correlation was used for statistical analysis.

Of 37 obese children, 19 children had bright liver on USG. The mean APRI was 0.16 (0.119). The mean Adiponectin was 4.04. Only one obese subject (5.0%) with bright liver had an APRI > 0.5. APRI was significantly correlated to alanine amino transferase (ALT) levels (*r* = 0.62), but not significantly correlated to BMI (*r* = 0.35) and adiponectin (*r* = 0.45). There was no correlation between BMI, ALT (*r* = 0.16), AST (*r* = 0.16), and adiponectin (*r* = 0.30).

This study suggest that obese children with fatty liver might have high APRI levels indicating the presence of liver fibrosis. However, there is no correlation between APRI, adiponectin and BMI.

